# Role of miR-155 and miR-103 in Oxidative Stress in Cardiovascular Disease: A Narrative Review

**DOI:** 10.3390/pathophysiology33030064

**Published:** 2026-08-25

**Authors:** Martina Cacciapuoti, Lucia Federica Stefanelli, Ilaria Caputo, Giulia Driussi, Monica Ceol, Giovanna Priante, Lorenzo A. Calò, Federico Nalesso

**Affiliations:** Nephrology, Dialysis and Transplantation Unit, Department of Medicine (DIMED), University of Padua, 35128 Padua, Italy; martina.cacciapuoti@phd.unipd.it (M.C.); luciafederica.stefanelli@unipd.it (L.F.S.); ilaria.caputo@unipd.it (I.C.); giulia.driussi@unipd.it (G.D.); monica.ceol@unipd.it (M.C.); giovanna.priante@unipd.it (G.P.); federico.nalesso@unipd.it (F.N.)

**Keywords:** miR-155, miR-103, oxidative stress, cardiovascular disease, hypertension, Gitelman’s syndrome

## Abstract

**Background/Objectives**: Oxidative stress is a major contributor to the pathogenesis of cardiovascular diseases, including hypertension, ischemic cardiomyopathy, and heart failure. MicroRNAs (miRNAs) have been extensively investigated in various contexts, and some of them have been identified to play a role in cardiovascular disease. This narrative review focuses on miR-103 and miR-155, two miRNAs implicated in the modulation of oxidative stress and cardiovascular remodeling. **Methods**: The following queries were used in PubMed since inception until May 2026: ((“miR-155” OR “microRNA-155” OR miR155) AND (“oxidative stress” OR ROS OR “reactive oxygen species”) AND (“cardiovascular disease” OR cardiovascular OR cardiac OR heart OR vascular)); ((“miR-103” OR “microRNA-103” OR miR103) AND (“oxidative stress” OR ROS OR “reactive oxygen species”) AND (“cardiovascular disease” OR cardiovascular OR cardiac OR heart OR vascular)). **Results**: A total of seven citations for miR-103 and 79 citations for miR-155 were identified. Reviews and papers about diseases other than those on cardiac/vascular involvement were excluded. miR-155 emerges as a potential regulator of inflammatory-redox signaling, whereas miR-103 appears more closely linked to cell fate and metabolic pathways. In both cases, available evidence supports a context-dependent role that challenges simplistic classification as pro- or antioxidant miRNAs. **Conclusions**: Available evidence suggests that both miR-103 and miR-155 are important regulators of oxidative stress-related pathways in cardiovascular disease. Nevertheless, the context-dependent effects observed across different cardiovascular disorders raise concerns regarding the safety of systemic miRNA modulation-based therapeutic strategies. Future studies should clarify the determinants of this context-dependent behavior and identify the specific conditions under which these miRNAs exert protective or harmful effects, which might pave the way for the development of miRNA-based therapeutic strategies targeting oxidative stress and cardiovascular remodeling.

## 1. Introduction

Oxidative stress is defined as the imbalance between the production and accumulation of pro-oxidant and antioxidant species [1,2]. Among reactive oxygen species (ROS), superoxide (O_2_^−^) is generated by several enzymatic sources, including NADPH oxidases, uncoupled nitric oxide synthase, xanthine oxidase, cytochrome P450, lipoxygenases, and cyclooxygenases. Due to its short half-life, O_2_^−^ is rapidly converted into hydrogen peroxide (H_2_O_2_) by superoxide dismutase 1 and 2 (SOD1/2), a more stable and membrane-permeable ROS, which can further generate highly reactive hydroxyl radicals. Besides acting as signaling molecules, ROS can react with nitric oxide to form toxic reactive species such as peroxynitrite. To counterbalance oxidative stress, cells rely on antioxidant defenses, including enzymatic scavengers, such as superoxide dismutase and glutathione peroxidase, as well as adaptive mechanisms such as autophagy, whose effects may be either protective or harmful depending on the intensity and duration of oxidative stress [2,3,4]. While oxidants are essential for pathogen killing, their excessive production induces tissue damage. Failure of the antioxidant armamentarium to successfully moderate prooxidant species results in detrimental effects that contribute to multiple pathophysiological processes, such as aging, cancer, cardiovascular disease, kidney disease, and autoimmune diseases [1,5,6,7,8,9]. Given the high oxygen and metabolic demands of the heart, the cardiovascular system is particularly susceptible to oxidative stress. Oxidative stress plays a critical role in the pathogenesis of multiple cardiovascular diseases, such as hypertension, fibrotic and ischemic cardiomyopathy, diabetes, and pre-eclampsia, and is interconnected with inflammation and senescence [10,11,12].

MicroRNAs (miRNAs) are short non-coding RNA molecules, typically 18–22 nucleotides in length. More than 1000 miRNA genes are encoded in the human genome. The biosynthesis starts with transcription by RNA polymerase II and synthesis of the hairpin structure. This precursor miRNA then binds to Ran-GTP and exportin 5 and is finally exported to the cell cytoplasm. MiRNA precursors are then transformed into double-stranded fragments of about 22 nucleotides by Dicer. The RNA-induced silencing complex (RISC) then integrates with the mature guided transcript (miRNA). This RISC–miRNA complex directly targets newly synthesized mRNAs to undergo biogenesis inhibition or mRNA degradation [13] by interacting with the 3′ untranslated region (3′UTR) of target mRNAs. Through this interaction, they can suppress protein synthesis or promote mRNA degradation, thereby modulating multiple cellular processes [14,15,16]. An individual miRNA can bind to multiple transcripts, and a single transcript can be bound by various miRNAs, determining an intricate and dynamic posttranscriptional regulation [17]. Recent literature has shown that miRNAs can play a multifaceted role in the interplay between oxidative stress and cardiovascular disease [8,9].

Among these, miR-103 (also reported as miR-103-3p) belongs to the miR-103/107 family and is located on human chromosome 5 [18]. It is expressed in various cell types and has been described as an oncogenic miRNA because of its ability to enhance cellular proliferation in several cancers, including non-small cell lung cancer, breast cancer, colon cancer, and squamous cell carcinoma. Beyond its role in tumor biology, the miR-103/107 family has also been associated with hypertensive nephropathy, cardiovascular remodeling, oxidative stress, and metabolic disorders [18,19,20].

MiR-155 (also reported as mir-155-5p) is a highly conserved single-stranded non-coding RNA transcribed from the B-cell integration cluster (BIC) gene located on chromosome 21. miR-155-5p derives from the 5′ arm of the precursor miRNA hairpin and is the more biologically active form. Although it is predominantly expressed in hematopoietic cells, its expression has also been detected in fibroblasts, epithelial and reproductive tissues, as well as in the central nervous system [14]. This miRNA participates in a wide range of biological functions, such as immune and inflammatory responses, regulation of apoptosis and cell proliferation, maintenance of vascular homeostasis, hypertension, cardiovascular remodeling, and cancer [14,21]. Owing to its pleiotropic functions, miR-155 has emerged as a particularly complex regulator in cardiovascular pathophysiology [14,21,22].

MiR-103 and miR-155 have both been studied in oxidative stress in cardiovascular disease, and recent evidence suggests they may exert either protective or detrimental effects depending on the pathological context. This narrative review intentionally focuses on miR-103 and miR-155, as they provide an opportunity to discuss the complexity of miRNA-mediated regulation of oxidative stress in cardiovascular diseases and summarizes the available evidence from in vivo/in vitro and human-based studies of the role played by miR-103 and miR-155 in oxidative stress in cardiovascular disease.

## 2. Methods

**Figure 1:**
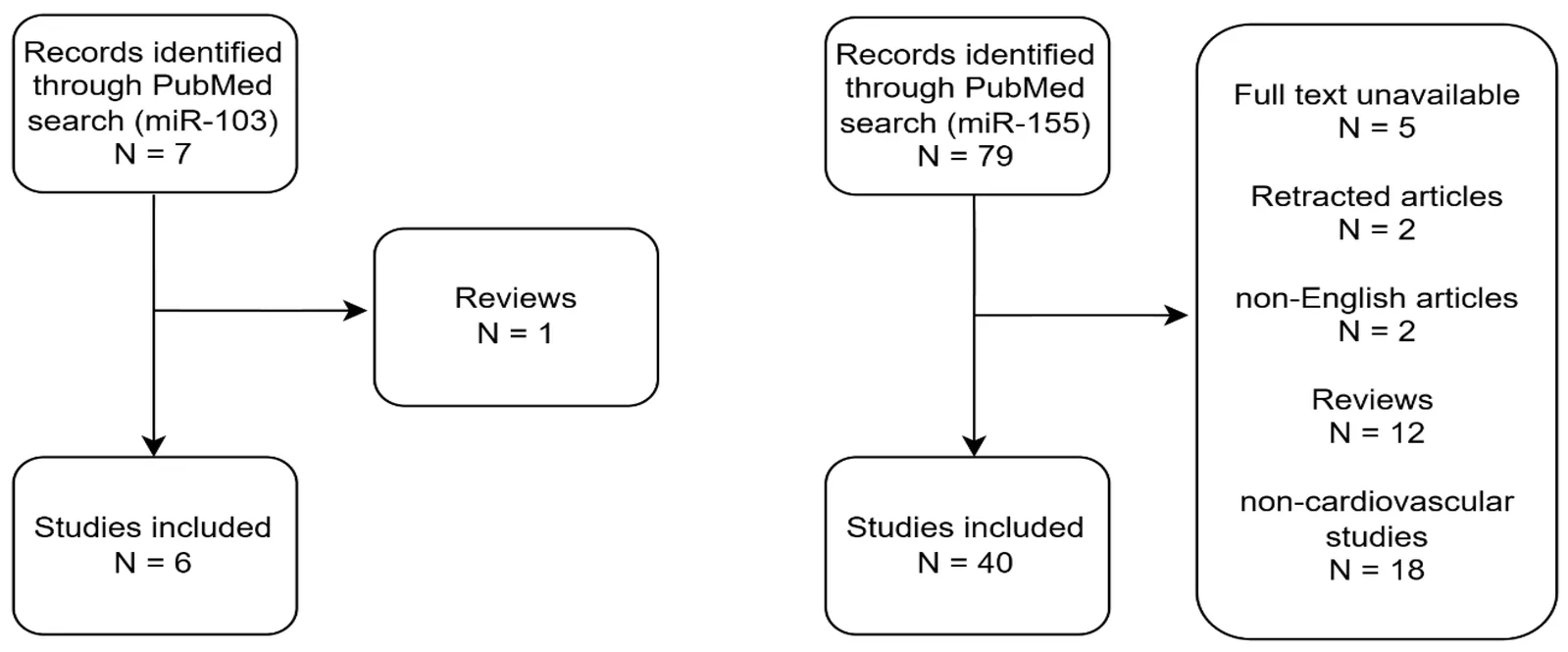
Flowcharts depicting study selection for miR-103 and miR-155.

The following queries were used in PubMed since inception until 11 May 2026: ((“miR-155” OR “microRNA-155” OR miR155) AND (“oxidative stress” OR ROS OR “reactive oxygen species”) AND (“cardiovascular disease” OR cardiovascular OR cardiac OR heart OR vascular)); ((“miR-103” OR “microRNA-103” OR miR103) AND (“oxidative stress” OR ROS OR “reactive oxygen species”) AND (“cardiovascular disease” OR cardiovascular OR cardiac OR heart OR vascular)). Study selection was performed by one reviewer and independently verified by a second reviewer. Any discrepancies in opinion were settled by discussion and consensus between the two reviewers. The search produced a total of 7 citations for miR-103 and 79 citations for miR-155, two of which were recently retracted. Access was limited for 5 papers. Reviews and papers about diseases other than those with cardiac, vascular, or cardiovascular involvement were excluded. Only articles in English were fully read (Figure 1, Table 1 and Table 2). It should be noted that some reports analyze the miR-103/107 family collectively because of the high sequence homology and overlapping biological functions of the miRNAs. Such cases have been acknowledged and specified in the text. 

## 3. Discussion

### 3.1. miR-103

MiR-103 has been particularly studied in the context of cardiac damage and function. Current evidence suggests a context-dependent role of miR-103 in cardiovascular oxidative stress, with both protective and detrimental effects being reported depending on the disease model and downstream molecular targets (Table 1).

**Table 1:** Summary of Evidence about miR-103 Role in Oxidative Stress in Cardiovascular Disease.

Author	Year	In Vitro/In Vivo Model	Pathway	Evidence Strength	Oxidative Stress Assessment	Main Study Limitations	Final Effect
Wang X. et al. [23]	2025	HL-1 mouse cardiomyocytes	The myocardium of diabetic mice showed upregulation of miR-103-3p High glucose enhanced ROS and MDA levels, decreased SOD, and increased miR-103-3p, which were reversed by orientin (an antioxidant agent) H19 overexpression inhibited high glucose-triggered ROS production in HL-1 cells, but miR-103-3p overexpression or ALDH2 depletion negated the effects of H19 overexpression High glucose → ↑ miR-103-3p → ↓ ALDH2 → ↓ PI3K/AKT → ↑ ROS	Mechanistic and intervention-based evidence	GSH, SOD, 4-HNE, MDA	Streptozocin-induced diabetic mice fed a high-fat diet may not fully recapitulate features of human diabetes mellitus type II; focused on a single signaling axis (H19/miR-103-3p/ALDH2/PI3K/AKT); no long-term safety/pharmacokinetic evaluation of orientin.	pro-apoptotic and pro-oxidant
Zhang et al. [24]	2022	Cardiomyocytes from transverse aortic constriction (TAC) mice	In TAC mice, miR-103-3p increases significantly and is associated with higher ROS levels, cardiac hypertrophy, increased ANP and beta-MHC, and worsening of cardiac function	Mechanistic evidence	DHE staining for ROS detection	RNA import assay in the mitochondria is validated in vitro but is supposed to be more complicated in vivo; small human sample size	pro-oxidant, pro-hypertrophic
		Heart tissue and plasma from HF patients and controls	lnccytb expression was significantly reduced in both plasma and heart failure tissue. Plasma lnccytb levels positively correlated with LVEF and inversely with NT-proBNP				
		Primary neonatal mouse cardiomyocytes	lnccytb acted as a competitive endogenous RNA via sponging miR-103-3p				
		HEK293T cells	miR-103-3p targets PTEN to promote isoprenaline-induced hypertrophy and ROS generation cardiac stress → ↓ lnccytb → ↑ miR-103-3p → ↓ PTEN → ↑ AKT → ↑ ROS, hypertrophy, heart failure				
Wang Y. et al. [25]	2020	Human coronary artery endothelial cells (HCAECs)	H2O2-induced oxidative stress downregulates miR-103 in a time-dependent manner pre-103 reduced the accumulation of autophagic ubiquitin-like p62 and LC3II proteins miR-103 inhibitor reduces cell survival rate in H_2_O_2_-induced oxidative stress, reducing p-mTOR/mTOR expression, thus inhibiting end-stage autophagy miR-103 inhibition increased the expression of BNIP3 miR-103 inhibition aggravates pyroptosis through the NLRP3 inflammasome and was associated with higher levels of IL-1β	Mechanistic evidence	H_2_O_2_-induced oxidative stress environment	In vitro HCAECs model only; acute (1 to 4 h) oxidative stress exposure; no in vivo/human validation	antioxidant
Xu et al. [20]	2015	Human umbilical vein endothelial cells (HUVECs)	H2O2 (5, 10, 25, 50, 100 and 200 µM) downregulated the expression of miR-103 in a time- and dose-dependent manner Cells transfected with miR-103 showed increased viability and lower intracellular ROS formation in an H2O2-induced oxidative stress environment by targeting BNIP3 miR-103 was upregulated following pretreatment with salidroside (an antioxidant agent) in an H2O2-induced oxidative stress	Mechanistic and intervention-based evidence	H_2_O_2_-induced oxidative stress environment	In vitro HUVEC model only; H_2_O_2_-induced oxidative stress; no in vivo/human validation; no rescue experiments.	antioxidant and anti-apoptotic
Wang J. et al. [26]	2015	H9c2 CELLS	High levels of H_2_O_2_ (500 µM) significantly increase the expression of miR-103/107 miR-103/107 are involved in H_2_O_2_-induced necrosis by targeting FADD	Mechanistic evidence	H_2_O_2_-induced oxidative stress environment	High-dose H_2_O_2_ in vitro model; no human validation.	pro-oxidant and pro-necrotizing
		mice model of I/R (ischemia/reperfusion)	Knockdown of miR103/107 decreases the expression levels of inflammatory cytokines TNF-alpha and interleukin-beta In the I/R mouse models, miR-103/107 antagomir administration resulted in a reduction in myocardial necrosis, reduced myocardial infarct sizes, and reduced plasma levels of the cardiac necrosis biomarker troponin T, without affecting myocardial apoptosis, reduced cardiac fibrosis, and ameliorated cardiac function				
Logan et al. [17]	2021	neonatal mice	Isoflurane and CO increase miR-103 levels vs. air	Observational evidence (expression profiling)	CO exposure	No gain-/loss-of-function validation of individual miRNAs; signaling pathways inferred from the previous literature rather than experimentally demonstrated	anti-apoptotic

Mechanistic evidence = when gain-/loss-of-function (mimics, inhibitors/antagomirs, knockdown/overexpression) and/or target validation (luciferase assays, rescue experiments) experiments were performed; pharmacologic evidence = when a pharmacologic treatment modifies miRNAs and phenotype, without direct causation demonstration; observational evidence = association studies (in vitro, in vivo models or patients), without direct miRNA manipulation. Abbreviations: 4-HNE: 4-hydroxynonenal; ALDH2: aldehyde dehydrogenase 2; BNIP3: BCL2 interacting protein 3; CO: carbon monoxide; DHE: dihydroethidium; FADD: Fas-associated protein with death domain; GSH: glutathione; H19: long non-coding RNA H19; H_2_O_2_: hydrogen peroxide; H9c2: rat embryonic cardiomyoblast cell line H9c2; HCAEC: human coronary artery endothelial cells; HEK293T: human embryonic kidney 293T cells; HF: heart failure; HUVECs: human umbilical vein endothelial cells; I/R: ischemia/reperfusion; IL-1β: interleukin-1 beta; LC3II: microtubule-associated protein 1 light chain 3-II; LVEF: left ventricular ejection fraction; MDA: malondialdehyde; mTOR: mammalian (or mechanistic) target of rapamycin; NT-proBNP: N-terminal pro-B-type natriuretic peptide; NLRP3: NOD-, LRR- and pyrin domain-containing protein 3; PI3K: phosphoinositide 3-kinase; PTEN: phosphatase and tensin homolog; ROS: reactive oxygen species; SOD: superoxide dismutase; TAC: transverse aortic constriction; TNF-α: tumor necrosis factor alpha; β-MHC: beta-myosin heavy chain.

**Figure 2:**
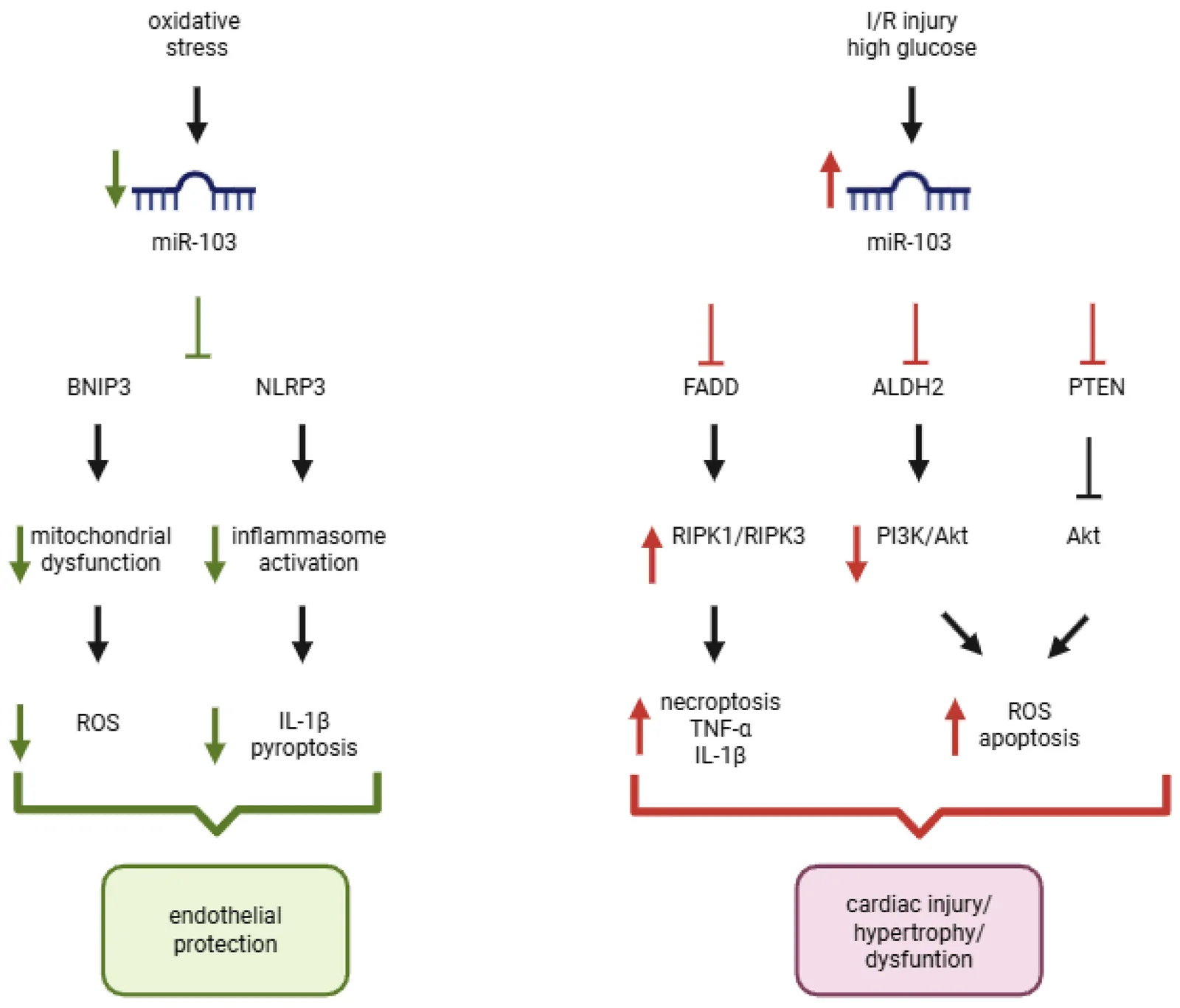
Context-dependent effects of miR-103 on cardiovascular oxidative stress. In endothelial cells exposed to oxidative stress, reduced miR-103 expression contributes to mitochondrial dysfunction and inflammasome activation through BNIP3 and NLRP3 signaling, whereas restoration of miR-103 attenuates ROS production, IL-1β release, and cell injury. Conversely, in pathological settings such as ischemia/reperfusion (I/R) injury, diabetic cardiomyopathy, and pressure overload-induced heart failure, increased miR-103/107 expression promotes inflammatory cell death, oxidative stress, and maladaptive remodeling by targeting FADD, ALDH2, and PTEN-dependent pathways. Collectively, current evidence indicates that miR-103 acts as a context-dependent regulator of oxidative stress and cell fate decisions in cardiovascular disease. Abbreviations: ALDH2: aldehyde dehydrogenase 2; BNIP3: BCL2 interacting protein 3; FADD: Fas-associated protein with death domain; IL-1β: interleukin-1 beta; I/R: ischemia/reperfusion; NLRP3: NOD-, LRR- and pyrin domain-containing protein 3; PTEN: phosphatase and tensin homolog; ROS: reactive oxygen species. Created in BioRender. Cacciapuoti, M. (2026) https://BioRender.com/1gyeils.

In cardiomyocytes from diabetic mice, high glucose increased miR-1033p levels together with ROS and reduced SOD. H19, a long non-coding RNA, worked as a sponge for miR-103-3p, thereby preventing the inhibition of ALDH2 (Aldehyde dehydrogenase 2), thus activating the PI3K/Akt protective pathway, ultimately resulting in reduced oxidative stress and apoptosis [23]. By reducing miR-103-3p, orientin, a flavonoid, exerted antioxidant effects [23]. Within the same pathway, in heart failure cardiomyocytes, miR-103 suppresses PTEN (phosphatase and tensin homolog), an inhibitor of AKT signaling [24], increasing ROS production and cardiac hypertrophy [24]. Interestingly, although miR-103 modulates different downstream targets across cardiovascular disorders, both studies converge on dysregulation of the PI3K/AKT signaling axis and oxidative stress pathways. In diabetic cardiomyopathy, miR-103-3p suppresses the antioxidant enzyme ALDH2, leading to impaired AKT-mediated cardioprotective signaling [23], whereas in pressure overload-induced heart failure, it targets PTEN, resulting in excessive AKT activation and maladaptive hypertrophic remodeling [24] (Figure 2).

MiR-103 has also been studied in oxidative stress environments. In in vitro models of induced oxidative stress, mechanistic evidence demonstrated that H_2_O_2_ downregulated the expression of miR-103 in a time-dependent manner. Nevertheless, cells transfected with miR-103 showed increased viability and lower intracellular ROS formation in an H2O2-induced oxidative stress environment by targeting BNIP3 (Bcl2/adenovirus E1B 19 kDa interacting protein 3), a mitochondrial pro-apoptotic protein involved in mitochondrial dysfunction, oxidative stress, and cell death [20,25]. In endothelial coronary artery cells, the same pathway demonstrated that miR-103 inhibition aggravates pyroptosis through the NLRP3 inflammasome and is associated with higher levels of IL-1β expression. Conversely, in vitro models (cardiomyocytes) of induced oxidative stress have shown that exposure to high levels of H_2_O_2_ (500 μM) significantly increases miR-103/107 levels, which target FADD (Fas-associated death domain protein), thereby decreasing its protein expression and impairing its protective effect on necroptosis by preventing the formation of the complex RIPK1/RIPK3 (Receptor-interacting serine/threonine-protein kinases 1 and 3) [26].

Interestingly, the protective effects observed in endothelial oxidative injury contrast with findings from ischemia/reperfusion (I/R) models, where miR-103/107 appear to promote inflammatory cell death pathways. These findings are supported by mechanistic evidence based on loss-of-function and rescue experiments. In fact, Wang et al. observed that miR-103/107 was increased in murine models of I/R and was associated with higher levels of TNF-α and IL-1β and with worsening cardiac function. Finally, miR103/107 antagomir administration significantly reduced the necrosis area induced by H_2_O_2_ in murine models of ischemia/reperfusion (I/R) [26].

Human-based studies evaluating miR-103 in cardiovascular diseases are sporadic and provide poor observational evidence. MiR-103 levels were found to be lower in heart failure patients compared to healthy volunteers [27], but oxidative stress parameters were not assessed. Additionally, in a cohort of patients with Gitelman syndrome, a model of reduced oxidative stress, plasmatic miR-103 levels were higher than in controls [28].

Beyond cardiovascular disease, miR-103/107 have also emerged as key regulators of metabolic homeostasis through modulation of insulin sensitivity, adipocyte differentiation, and fatty acid β-oxidation via Caveolin-1-dependent pathways. These findings lead to the development of anti-miR-103/107-based treatment strategies for type 2 diabetes mellitus with Non-alcoholic Fatty Liver Disease (NAFLD)—dosed in a first-in-human phase I study by Regulus’s collaboration partner AstraZeneca—and non-alcoholic steatohepatitis (NASH) [28,29,30]. Nevertheless, whether modulation of miR-103 and miR-103/107 may represent a viable therapeutic strategy in cardiovascular diseases has not been investigated yet, given the context-dependent effects reported across different models of oxidative stress and cardiac injury. For now, no specific molecule targeting this miRNA is being studied as a potential therapy for cardiovascular diseases.

### 3.2. miR-155

Current findings do not support a univocal role for miR-155 in cardiovascular oxidative stress. Rather, its effects appear to vary according to the disease context, cellular environment, and downstream molecular targets involved (Table 2).

**Table 2:** Summary of Evidence about miR-155 Role in Oxidative Stress in Cardiovascular Disease.

Author	Year	In Vitro/In Vivo/Human-Based Studies	Pathway	Evidence Strength	Oxidative Stress Assessment	Main Study Limitations	Final Effect
Ge et al. [31]	2026	Cardiomyocytes from mice	miR-155-5p is associated with ferroptosis and is increased in cells after incubation with H2O2; miR-155-5p targets NFE2L2, thereby inhibiting the promotion of expression of protective and antioxidative genes	Mechanistic evidence	MDA, 4-HNE, NADP/NADPH, ferro, GPX4, Ptgs2 and antioxidant genes Nqo1, HO-1, Fth1 e Slc7a11	Only acute assessment (24 h), no human validation.	pro-oxidant and pro-ferroptotic
DuPont et al. [32]	2016	Smooth muscle cells-MR-KO mice	miR-155 was downregulated with aging and associated with an increase in MR (mineralocorticoid receptor) expression miR-155 restoration reduced Cav1.2 and Agtr1 expression, attenuating AT2-induced vasoconstriction and oxidative stress	Mechanistic evidence	DHE staining of mesenteric arteries after Ang II stimulation	DHE staining for oxidative stress evaluation is not completely specific; small patient sample size.	antioxidant, antihypertensive
		HEK293 cells	MR repressed the miR-155 promoter in a ligand-independent way				
		Elderly hypertensive patients	Baseline serum miR-155 levels and treatment-induced increases in miR-155 predicted the blood pressure–lowering response to MR antagonism (eplerenone)				
He J. et al. [33]	2025	Bone marrow derived cells M1 polarized; endothelial cells from mouse aorta	Endothelial cells co-cultured with M1 exosomes carrying high levels of miR-155 had a pro-senescence effect by targeting SOCS1, activating JAK2/STAT3, and increasing ROS production in endothelial cells miR-155-5p mimic decreases SOCS1 but does not target BACH1 or IKBKE	Mechanistic evidence	MitoSOX, flow cytometry, mitochondrial respiration	Exogenous exosome administration may not fully recapitulate physiological conditions; no human validation.	pro-oxidant, pro-apoptotic, and pro-senescent
Zhang Y. et al. [34]	2024	HUVECs and HEK293 cells	miR-155-5p mimic increases ROS levels and decreases a-SMA and Vim; miR-155-5p inhibitor increases SIRT1, Nrf2 and HO-1 expression	Mechanistic evidence	ROS, mitochondrial ROS (MitoSOX), mitochondrial membrane potential (JC-1)	No in vivo rescue experiment	pro-oxidant
Tong et al. [35]	2023	Wistar Kyoto rats (WKY) and spontaneously hypertensive rats (SHR)	miR-155-5p is lower in primary VSMCs from spontaneously hypertensive rats than in WKY rats miR-155-5p exogenous administration mitigates oxidative stress (NOX2 and NOX4 protein expression and activity and decrease in ROS levels) in VSMCs from SHR miR-155-5p overexpression inhibited BACH1 in VSMCs from both SHR and WKY rats, with a reduction in oxidative stress and cell migration; with an inverse pattern with mIR-155-5p inhibitor	Mechanistic evidence	ROS measured by DCFH-DA fluorescence assay, NOX2 and NOX4 expression/activity.	Single-target focus; no demonstration of HO-1 in the downstream signaling of BACH-1; no in vivo study.	antioxidant
Zhao M. et al. [36]	2024	Ex vivo primary vascular endothelial cells from thoracic aortas of rat offspring	Hypoxic offspring-derived endothelial cells showed higher levels of miR-155-5p; miR-155-5p mimic increased miR-155-5p expression; miR-155-5p inhibitor reduced ROS production in offspring endothelial cells and reduced NO synthesis	Mechanistic evidence	DHE staining, NO release and intracellular Ca levels	No rescue experiment or direct target validation; only male rat offspring investigated; no in vivo modulation of miR-155-5pd.	pro-oxidant
Frati et al. [37]	2020	Smokers	Plasmatic miR-155 is significantly increased shortly after smoking	Mechanistic evidence in cells; observational (associative) evidence in humans	H2O2 production and NO metabolites nitrite and nitrate detected with specific kits	Choice of miR-155 based on the previous literature (no unbiased screening of miRNAs); only acute effects of smoke evaluated; small sample size without correction for multiple testing.	pro-oxidant and vasostrictive
		HUVECs	miR-155 accumulates in medium after exposure to cigarette smoke condensate anti-miR-155 attenuated condensate smoke-induced angiogenesis, oxidative stress, and NO production miR-155 mimic decreased cell viability, impaired capillary network formation and impaired capillary network, reduced VEGF and eNOS				pro-oxidant
He et al. [38]	2020	Human aortic smooth muscle cells HASMCs	Indoxyl-sulfate (uremic toxin) increases miR-155-5p expression with a corresponding increase in ROS and decline in Matrix Gla protein (MGP), which is reversed by NFκB inhibition	Mechanistic evidence	ROS were detected with DCFH-DA probes	No in vivo miR-155-5p functional validation; direct MGP targeting not validated in this study.	pro-oxidant and pro-inflammatory
Wang X. et al. [39]	2022	Endothelial cells from two kidneys, one clip, hypertensive rats	miR-155-5p expression was higher in hypertensive rats vs. control rats and accompanied by a decrease in eNOS and an increase in oxidative stress and was reversed by t-AUB treatment; results were confirmed by miR-155-5p inhibitor and mimics.	Mechanistic evidence	DHE staining for ROS detection; MitoSOX for mitochondrial ROS; concentration of nitrate and nitrite for NO evaluation	No in vivo genetic validation; mechanistic pathways remain partly inferential.	pro-oxidant
Wu N. et al. [40]	2020	Spontaneous hypertensive rats (SHR) and Wistar-Kyoto rats (WKY)	miR-155-5p mimic inhibited ACE, NOX2, IL-1β, TNF-α expression in VSMCs of spontaneous hypertensive rats Exogenous Ang II increased miR-155-5p expression in WKY rats but not in SHR	Mechanistic evidence	DHE staining for ROS detecton and NOX2 activity	No human validation	antioxidant, anti-inflammatory
Jia C. et al. [41]	2017	Hearts from ovariectomized diabetic mice	MIr-155 expression was higher in diabetic ovariectomized (OVX) mice than in diabetic mice not ovariectomized, together with increased M1 polarization	Mechanistic evidence	M1 polarization	disease model (OVX + STZ diabetic mice) may not fully. recapitulate human cardiometabolic disease; no human validation.	pro-inflammatory, pro-oxidant
		RAW264.7 cells	AuNP-mediated miR155 antagonist delivery promotes M2 polarization with a reduction in IL-1β and an increase in IL-10, a restoration of cardiac function, and an increase in vascular density				
Liu Y. et al. [42]	2015	Human brain microvessel endothelial cells HBMECs	Silencing of miR-155 decreases apoptosis and ROS production, while promoting NO generation in both vehicle- and ox-LDL-treated cells via the PI3K/Akt signaling pathway	Mechanistic evidence	DHE staining for ROS detection, concentration of nitrate and nitrite for NO evaluation	No direct validation of miR-155 target genes (no luciferase or rescue experiments); no in vivo/human validation.	pro-oxidant and pro-apoptotic
Liu J. et al. [43]	2011	Cardiomyocyte progenitor cells	miR-155 inhibits oxidative-stress-induced necrosis by targeting RIP-1	Mechanistic evidence	H2O2-induced oxidative stress, cell viability evaluation	Oxidative stress induced by H_2_O_2_, which only partially mimics the ischemic myocardial environment; No in vivo validation; Only miR-155 overexpression was protective, whereas inhibition of endogenous miR-155 did not increase necrosis, suggesting uncertain physiological relevance.	anti-necrotic
Wang F. et al. [44]	2022	Mouse model of myocardial fibrosis	Apigenin reduces oxidative stress and miR-155-5p expression in isoproterenol-induced myocardial fibrotic mice	Mechanistic evidence and pharmacologic evidence	MDA, SOD, and GSH-PX (glutathione peroxidase) measurements	no miR-155 overexpression (gain-of-function) rescue experiments to demonstrate necessity/sufficiency of miR-155 for apigenin’s effects.	pro-oxidant and profibrotic
		CFs cell line	miR-155-5p inhibitor reduces the TGF-β1/smad miR-155-5p mimic significantly reduces HIF-1α				
Sun et al. [45]	2016	Human aortic VSMCs	Salusin-beta (a stimulator of the progression of atherosclerosis) increased miR155 expression; miR-155 inhibition prevented Salusin-beta effects on ACAT-1 and VCAM-1 expression, p65-NFkB nuclear translocation, lipid accumulation, monocyte adhesion, and ROS production in VSMCs	Mechanistic evidence	DHE staining for ROS detection	No in vivo or clinical validation	pro-oxidant and pro-atherogenic
Yang et al. [46]	2015	Mice femoral arteries	Injured vessels in miR-155-/- mice showed decreased proliferation; injured arteries showed higher expression of miR-155 vs. uninjured arteries miR-155 down-regulates MST2 which competes with MEK for RAF-1 binding, resulting in ERK1/2 activation and ultimately NFκB and p47phox activation	Mechanistic evidence	NF-κB and p47phox expression	Mechanism focused mainly on one target (MST2); no human validation.	pro-inflammatory and pro-oxidant
Tian et al. [47]	2014	ApoE-/- mice	The level of miR-155 in the plasma of atherosclerotic mice is increased, and oxLDL effectively induces the expression of miR-155 in macrophages. miR-155 mediates oxLDL-induced lipid uptake and reactive oxygen species (ROS) production of macrophages by targeting HBP1. Repression of HBP1 by miR-155 transforms macrophages into foam cells	Mechanistic evidence	ROS production assessed with DCFH-DA	Limited human validation (expression only; no functional human experiments); mechanism focused on foam cell formation/early atherogenesis, limiting generalizability to advanced disease.	pro-oxidant; pro-atherosclerotic
		Patients with coronary heart disease	miR-155 expression is up-regulated in CD14 + monocytes from patients with coronary heart disease miR-155 inhibition decreases lipid loading in macrophages and reduces atherosclerotic plaques in ApoE-/- mice and is associated with a reduction in TNF-alpha and IL-6 expression				
Kim J. et al. [48]	2017	HUVECs	Aspirin inhibits ROS-mediated vasoconstriction, inflammation, and endothelial dysfunction by down-regulating miR-155 in pre-eclampsia	Mechanistic and pharmacologic evidence	DAF-FM diacetate for NO detection, DCFH-DA for ROS detection	No in vivo validation	pro-oxidant and pro-inflammatory
Kim TH et al. [49]	2020	HUVECs	Korean Red ginseng extract (KRGE) induced HO-1 and inhibited NFkB-dependent miR-155-5p biogenesis with the downregulation of eNOS miR-155-5p levels were increased in senescent HUVECs vs. young cells; and the increase was reversed by KRGE or NFκB inhibitor	Mechanistic evidence	DAF-FM diacetate for NO detection, DHE staining for ROS detection	No in vivo investigation	pro-oxidant and pro-inflammatory
Song et al. [50]	2021	HUVECs and VSMCs	miR-155 expression decreased in extravesicles from HUVECs treated with AT II, compared to the control and LSW treatment, and was associated with an increase in oxidative stress and inflammatory cytokines; LSW treatment reduced oxidative stress and increased miR-155	Observational (associative) and indirect pharmacologic evidence	DHE staining for ROS detection	Mechanistic conclusions rely mainly on transcriptomic and pathway enrichment analyses without validation of downstream miR-155 targets; no gain-/loss-of-function studies; DHE staining for oxidative stress evaluation is not completely specific.	antioxidant (indirectly)
Xiong et al. [51]	2015	ApoE -/- mice	Shexiang Tongxin dropping pill (STDP) treatment is associated with a significant reduction in MDA, ox-LDL, increased SOD, reduced ROS, and pro-inflammatory cytokines, and with a significant reduction in miR-155-5p expression in ApoE -/- mice aorta	Observational (associative) and indirect pharmacologic evidence	DHE staining for ROS detection	No gain-/loss-of-function experiments; no validation of downstream miR-155-5p targets or signaling pathways; multi-component herbal formulation, making it impossible to attribute the observed effects specifically to miR-155 modulation or to identify the active compound(s).	pro-oxidant and pro-inflammatory (indirectly)
Cai et al. [52]	2020	TNF-α -/- mice	TNF-α KO DOCA/Salt-hypertensive mice showed reduced oxidative stress, increased eNOS expression, and inhibited miR-155 expression in the aorta	Observational (associative) evidence	DHE staining for ROS detection, p22phox, gp91 phox, eNOS	No direct functional validation of miR-155; causal role inferred from prior studies; no human investigation.	pro-oxidant and pro-inflammatory (indirectly)
Harrison- Bernard et al. [53]	2024	Dahl salt-sensitive (DS) and spontaneously hypertensive rats (SHR)	DS-high salt rats showed significantly higher levels of aortic and kidney AT1R, p-JAK/JAK2, p-MYPT1/MYPT1, Arhgef, and proteinuria, lower kidney and serum klotho, and lower serum and aortic miR-155 vs. DS-low salt rats. SHR showed higher levels of miR-155, with no higher levels of AT1R.	Observational (associative) evidence	p-MYPT1/MYPT1	No gain-/loss-of-function experiments or direct target validation; the relationship between miR-155 expression and the α-klotho/AT1R/TNF-α axis remains associative rather than causal.	antioxidant
Costantino et al. [54]	2016	Diabetic mice	miR-155 (among those involved in oxidative stress) was reduced in diabetic mice, and the impairment persisted despite normalization of blood glucose levels	Observational (associative) evidence	Oxidative stress pathways identified by ingenuity pathway analysis	Oxidative stress involvement inferred by pathway analysis (IPA), not directly measured; no gain-/loss-of-function experiments; No causal experiments linking miR-155 to diabetic cardiac oxidative stress.	pro-oxidant (indirectly)
Munoz-Pacheco et al. [55]	2012	THP-1 cells (human monocytic cell line)	Phorbol-12-myristate-13-acetate (PMA) treated THP-1 cells showed increased levels of ROS and miR-155; ezetimibe-induced inhibition of THP-1 cell differentiation was associated with the down-regulation of the expression of miR-155, miR-222, miR-424, and miR-503; MAP Kinase and NF-κB pathways, as well as oxidative stress, are involved in this effect	Indirect pharmacological evidence	DHE staining for ROS detection	PMA-induced differentiation may not fully recapitulate atherosclerosis in vivo; no gain-/loss-of-function experiments or direct target validation for miR-155; No in vivo or clinical validation.	pro-oxidant (indirectly)
Santana et al. [56]	2020	HUVECs and PBMCs	Hydroxyurea reduces intracellular ROS and increases antioxidant enzymes (SOD1, GSR, GPX1), contemporarily up-regulating miR-155-5p expression	Observational associative evidence	DCFH-DA for ROS detection, NO concentration	No direct assessment of miR-155 expression (only bioinformatic prediction), association inferred from transcriptomic analysis, no functional validation; no gain-/loss-of-function studies.	antioxidant (indirectly)
Nguyen et al. [57]	2021	Human VSMCs	miR-155-5p (both intracellular and exosomal) decreases in senescent cells and is associated with elevated oxidative stress	Observational evidence	genes PCR evaluation: CAT, SOD1, SOD2, GPX1, GPX4, GSTP1, IL-6, IL-8, and MCP	No gain-/loss-of-function analysis; target pathways inferred from available bioinformatic datasets, no functional validation; association between miR-155 downregulation and oxidative stress is correlative; no in vivo model.	pro-oxidant (indirectly)
Khedr et al. [58]	2024	Human-based studies (metabolic syndrome patients)	miR-155-3p levels decreased after 6 months of green coffee treatment together with inflammation and oxidative stress parameters; miR-155-3p positively correlates with HbA1c, glucose levels, and HOMA-IR (Homeostatic Model Assessment for Insulin Resistance)	Observational (associative) evidence	MDA	No functional validation	antioxidant (indirectly)
Duisenbek et al. [59]	2024	Human-based study (diabetic patients)	miR-155-5p levels are higher in diabetic patients than in controls and are positively associated with HbA1c and glucose levels; predicts diabetes in obese subjects along with glutathione peroxidase and lipid peroxidation levels	Observational (associative) evidence	Advanced oxidation protein products, lipid peroxidation, nitric oxide; antioxidant enzyme activities (SOD, CAT, G6PD)	No functional validation	pro-oxidant
Moawad et al. [60]	2024	Human-based study (coronary heart disease patients)	Coronary heart disease patients showed higher levels of miR-155-5p together with higher MDA and lower SOD	Observational (associative) evidence	MDA, SOD	No functional validation	pro-oxidant (indirectly)
Alizadeh Saghati et al. [61]	2024	Human cardiac tissue	Hearts of patients with COVID-19 present increased activation of ferroptosis and oxidative stress; miR-155-5p is predicted to be involved in this pathway	In silico observational evidence based on bioinformatic tools	Bioinformatic networks associated with oxidative stress	No experimental validation (hypothetical role of miR-155).	pro-oxidant
Kelly et al. [62]	2011	Friedreich’s ataxia patients	Polymorphism rs5186—which increases expression of AGTR1 by altering the binding site for miR-155—is associated with cardiac hypertrophy (to which oxidative stress contributes)	Observational evidence	Indirect link between AT1R signaling and oxidative stress	Small sample size; single SNP evaluation; absence of comprehensive clinical data in the control population.	anti-hypertrophic (indirectly)
Kim et al. [63]	2015	Smokers vs. non-smokers	Male smokers showed 3-fold higher levels of miR-155 in HDL; 8 weeks of vitamin C reduced miR-155 levels in HDL in male smokers and non-smokers	Observational (associative) and indirect pharmacologic evidence	OxLDL, oxHDL, MDA, serum antioxidant capacity	No gain-/loss-of-function experiments; small sample size	pro-oxidant (indirectly)
Chen H. et al. [64]	2019	Endothelial cells	miR-155-5p inhibition promotes endothelial cells proliferation and reduces SOD expression; miR-155-5p regulates autophagy via decreasing the expression of ATG5	Mechanistic evidence	SOD	In vitro H_2_O_2_-treated HUVEC model with no in vivo or clinical validation; oxidative stress model does not fully reproduce the vascular microenvironment.	pro-oxidant and anti-proliferative
Liu et al. [65]	2020	miR-155 knockout transcriptomic datasets and ApoE-/- mouse data (bioinformatic analysis)	miR-155 deficiency reduced atherosclerosis in ApoE-/- mice and altered innate immune and ROS-related signaling pathways	Observational evidence (bioinformatic analysis)	ROS-related signaling pathways	Based primarily on bioinformatic analysis of public microarray datasets; no original experimental validation (in vitro/in vivo), no direct target validation, and heterogeneous datasets from different tissues/cell types.	pro-inflammatory; indirect regulator of oxidative stress
Constantin et al. [66]	2022	Human-induced pluripotent stem cell-derived cardiomyocytes hiPSC-CMs and bone marrow-derived stem cells BMMSC	No difference in miR-155-5p expression in the extracellular vesicles of the two types of cells, nor vs. treatment with ATII and TGF-β	Observational (associative) findings- no significant findings	DCFH-DA for ROS detection	No functional investigations	not statistically significant
Hefti et al. [67]	2014	Hearts from Down syndrome and non-Down Syndrome donors	Difference in expression of miR-155 and BACH1	Observational findings- non-significant findings	Indirect link between BACH1 and oxidative stress	Small sample size (pilot study); no gain-/loss-of function experiments	not statistically significant
Jia et al. [68]	2014	ApoE-/- mice	NR1 treatment in ApoE-/- mice induced higher expression of SOD, GSH, reduced ROS, and pro-inflammatory cytokines, along with a reduction in miR-155-5p (not statistically significant)	Observational findings—non-significant findings	The serum concentrations of SOD, GSH, and MDH, and oxLDL levels	No gain-/loss-of-function experiments	not statistically significant
Liu D et al. [69]	2014	Patients with intracranial aneurysms	miRNA profile differed in intracranial aneurysms vs. superficial temporal arteries (miR-155 not significant)	Observational findings—non-significant findings	Oxidative stress was only inferred by bioinformatic pathway enrichment of predicted/validated target genes.	Small sample size; no high-throughput sequencing for miRNAs	not statistically significant
Witvrouwen et al. [70]	2021	Pre-eclampsia and healthy pregnant women	miR-155 was not significantly different between groups	Observational findings—non-significant findings	Superoxide levels	Small sample size	not statistically significant

Mechanistic evidence = when gain-/loss-of-function (mimics, inhibitors/antagomirs, knockdown/overexpression) and/or target validation (luciferase assays, rescue experiments) experiments were performed; pharmacologic evidence = when a pharmacologic treatment modifies miRNAs and phenotype, without direct causation demonstration; observational evidence = association studies (in vitro, in vivo models or patients), without direct miRNA manipulation. Abbreviations: HNE: 4-hydroxynonenal; ACE: angiotensin-converting enzyme; AGTR1 (AT1R): angiotensin II receptor type 1; Akt (AKT): protein kinase B; ApoE-/-: apolipoprotein E knockout; ATG5: autophagy-related protein 5; ATII (Ang II): angiotensin II; AuNP: gold nanoparticle; BACH1: BTB and CNC homology 1; BMMSC: bone marrow-derived mesenchymal stem cells; CAT: catalase; Cav1.2: L-type voltage-dependent calcium channel subunit alpha-1C (CACNA1C); CFs: cardiac fibroblasts; COVID-19: coronavirus disease 2019; DCFH-DA: 2′,7′-dichlorodihydrofluorescein diacetate; DHE: dihydroethidium; DOCA: deoxycorticosterone acetate; DS: Dahl salt-sensitive; eNOS: endothelial nitric oxide synthase; ERK1/2: extracellular signal-regulated kinases 1 and 2; Ferro: ferroptosis-related markers; Fth1: ferritin heavy chain 1; G6PD: glucose-6-phosphate dehydrogenase; GPX1: glutathione peroxidase 1; GPX4: glutathione peroxidase 4; GSH: reduced glutathione; GSH-PX: glutathione peroxidase; GSR: glutathione reductase; GSTP1: glutathione S-transferase Pi 1; HASMCs: human aortic smooth muscle cells; HBMECs: human brain microvascular endothelial cells; HBP1: HMG-box transcription factor 1; HbA1c: glycated hemoglobin; HEK293: human embryonic kidney 293 cells; HEK293T: human embryonic kidney 293T cells; HIF-1α: hypoxia-inducible factor 1 alpha; hiPSC-CMs: human induced pluripotent stem cell-derived cardiomyocytes; HO-1: heme oxygenase-1; HOMA-IR: homeostatic model assessment for insulin resistance; HUVECs: human umbilical vein endothelial cells; IKBKE: inhibitor of nuclear factor kappa-B kinase subunit epsilon; IL-1β: interleukin-1 beta; IL-6: interleukin-6; IL-8: interleukin-8; KRGE: Korean red ginseng extract; MCP-1: monocyte chemoattractant protein-1; MDA: malondialdehyde; MEK: mitogen-activated protein kinase; MGP: matrix Gla protein; MitoSOX: MitoSOX red mitochondrial superoxide indicator; MR: mineralocorticoid receptor; MST2: mammalian sterile 20-like kinase 2 (STK3); MYPT1: myosin phosphatase target subunit 1; NADP/NADPH: nicotinamide adenine dinucleotide phosphate (oxidized/reduced forms); NF-κB: nuclear factor kappa B; NFE2L2 (NRF2): nuclear factor erythroid 2-related factor 2; NLRP3: NOD-, LRR- and pyrin domain-containing protein 3; NO: nitric oxide; NOX2: NADPH oxidase 2; NOX4: NADPH oxidase 4; Nqo1: NAD(P)H quinone dehydrogenase 1; oxHDL: oxidized high-density lipoprotein; oxLDL: oxidized low-density lipoprotein; OVX: ovariectomized; p22phox: NADPH oxidase subunit p22phox; p47phox: NADPH oxidase subunit p47phox; PBMCs: peripheral blood mononuclear cells; PI3K: phosphoinositide 3-kinase; PMA: phorbol 12-myristate 13-acetate; Ptgs2: prostaglandin-endoperoxide synthase 2 (COX-2); RAW264.7: murine macrophage cell line RAW264.7; RIPK1 (RIP-1): receptor-interacting serine/threonine-protein kinase 1; ROS: reactive oxygen species; SHR: spontaneously hypertensive rats; Slc7a11: solute carrier family 7 member 11 (xCT cystine/glutamate antiporter); SOCS1: suppressor of cytokine signaling 1; SOD: superoxide dismutase; SOD1: Cu/Zn-superoxide dismutase; SOD2: Mn-superoxide dismutase; STDP: Shexiang Tongxin Dropping Pill; STZ: streptozotocin; TGF-β1: transforming growth factor beta 1; THP-1: human monocytic leukemia cell line THP-1; TNF-α: tumor necrosis factor alpha; VCAM-1: vascular cell adhesion molecule-1; VEGF: vascular endothelial growth factor; Vim: vimentin; VSMCs: vascular smooth muscle cells; WKY: Wistar Kyoto rats.

**Figure 3:**
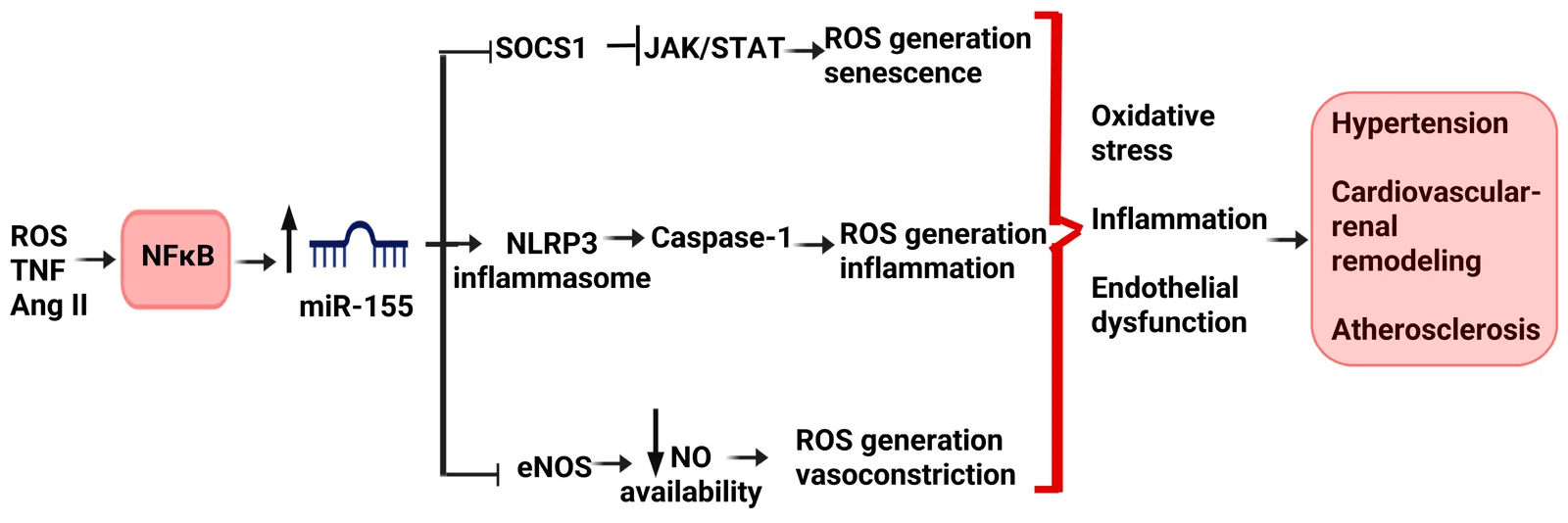
Proposed NFκB-dependent pro-oxidant signaling mediated by miR-155 in cardiovascular disease. Oxidative and inflammatory stimuli, including ROS, TNF-α, and Ang II, activate NFκB, which promotes miR-155 expression. Increased miR-155 contributes to oxidative stress and vascular injury through multiple mechanisms, including suppression of SOCS1 with subsequent activation of JAK/STAT signaling, stimulation of the NLRP3 inflammasome pathway, and inhibition of eNOS, resulting in reduced nitric oxide bioavailability. Collectively, these pathways promote inflammation, endothelial dysfunction, cellular senescence, and ROS generation, ultimately contributing to hypertension, vascular remodeling, and atherosclerosis. Abbreviation: Ang II: angiotensin II; eNOS: endothelial nitric oxide synthase; JAK/STAT: Janus kinase/signal transducer and activator of transcription; miR-155: microRNA-155; NFκB: nuclear factor kappa B; NO: nitric oxide; ROS: reactive oxygen species; SOCS1: suppressor of cytokine signaling 1; TNF-α: tumor necrosis factor alpha. Created in BioRender. Cacciapuoti, M. (2026) https://BioRender.com/14u2rob.

One of the main pathways linking miR-155 and oxidative stress in cardiovascular disease is that of NFκB. In fact, miR-155 biogenesis can be induced by NFκB, but miR-155 targets can also lead to NFκB activation, thereby contributing to oxidative stress and inflammation (Figure 3).

MiR-155 was broadly studied in atherogenesis [15]. MiR-155 expression was found to be induced by oxLDL (but not native LDL) in macrophages, by worsening oxidative stress conditions. Moreover, miR-155 promoted ROS production in oxLDL-induced Raw264.7 cells [47]. These findings were also supported by two more studies conducted in endothelial cells, where anti-miR-155-5p attenuated oxidative stress (demonstrated by SOD expression reduction), and anti-miR-155-5p increased NO production, respectively, thereby contrasting endothelial dysfunction [37,64]. MiR-155 was demonstrated to promote vascular smooth muscle cell proliferation by inhibiting MST-2, which was identified as a target of miR-155, thus favoring inflammation and oxidative stress [46]. In human VSMCs (vascular smooth muscle cells), miR-155 played a proatherogenic role in studies based on pharmacologic interventions: miR-155 inhibition abolished the effects of salusin-β on inflammation, monocyte adhesion, and ROS production in VSMCs [45]. In ApoE-/- mice, treatment with Shexiang Tongxin dropping pill (STDP)—a traditional Chinese medicine treatment for angina pectoris—was associated with a reduction in oxidative stress and inflammation and was accompanied by a decrease in miR-155-5p levels [51].

In endothelial cells from aortas derived from gestational hypoxia offspring rats, a model of pre-eclampsia, miR-155-5p levels were elevated and associated with increased expression of NADPH oxidase 2 and ROS generation, as well as impaired NO synthesis. In this context, a miR-155-5p inhibitor reduced ROS production, suggesting a causal role in pre-eclampsia pathogenesis [36]. Aspirin is a preventative strategy in pre-eclampsia. In HUVECs, aspirin was reported to inhibit the TNF-α induced miR-155 biogenesis by suppressing NFκB activation, thereby reducing endothelial dysfunction, vasoconstriction, and inflammation [48]. In HUVECs, Korean red ginseng extract delayed senescence, reduced oxidative stress, and blocked NFκB-dependent miR-155-5p biogenesis, resulting in increased eNOS expression [49], and the same pathway was confirmed in hypertensive rats [39]. In indoxyl sulfate-treated human aortic cells mimicking uremic vascular calcifications, NFκB activation induced miR-155-5p biogenesis along with an increase in Matrix Gla Protein and an increase in ROS production, both reversed by NFκB inhibition [38]. Finally, miR-155-5p was elevated in hypertensive mice’ renal arteries and endothelial cells and was accompanied by increased ROS, NFκB activation, and decreased eNOS [39].

Regarding cardiomyopathies in cardiomyocytes transferred with extracellular vesicles derived from an ischemia–reperfusion (IR) environment, the expression of miR-155-5p was significantly increased and contributed to ferroptosis by targeting and inhibiting the Nfe2l2 pathway, which regulates the expression of cytoprotective and antioxidative genes [31]. Addition of miR-155-5p mimics also facilitated oxidative stress with a reduction in NADPH levels and an increase in MDA production [31]. In mice models of fibrotic cardiomyopathy, miR-155 levels were associated with oxidative stress and activation of the HIF-1-α and TGF-β signaling pathways, while treatment with apigenin, an antioxidant flavonoid molecule, decreased miR-155-5p levels [44]. Finally, ferroptosis and oxidative stress are pathological mechanisms involved in cardiac damage in SARS-CoV-2 infection, and observational evidence based on bioinformatic analysis suggested that miR-155-5p may play a role too [61].

Oxidative stress triggered by hyperglycemia, insulin resistance, and hyperlipidemia is a pivotal contributor to endothelial dysfunction, which ultimately leads to high-mortality-burdened macro- and microvascular complications in diabetes [11]. Research by Zhang et al. observed that in HUVECs exposed to a high-glucose environment, miR-155 promoted ROS generation by targeting the SIRT1/Nrf2/HO-1 pathway and induced epithelial–mesenchymal transition, suggesting a role of this miRNA in the difficult wound-healing process in diabetes [34]. In ovariectomized diabetic mice, observational results showed that miR-155 expression was enhanced in the macrophages along with an increase in ROS, cell apoptosis, cardiac hypertrophy, and fibrosis in the heart [41]. The administration of antagomiR-155 covalently conjugated with a gold nanoparticle (AuNP) successfully delivered the nucleic acids into the macrophages, increasing M2 macrophages over M1 proliferation and reducing inflammation and cardiac damage, making it a promising strategy for improving cardiac function [41].

MiR-155 was also identified to intervene in the SOCS1 signaling pathway within the context of diabetic damage and aging. SOCS1 was confirmed to be a target of miR-155 also in endothelial cells, leading to cell cycle arrest (by increasing p21) and ROS production, ultimately resulting in a pro-senescence effect [33]. Another study, though conducted in diabetic kidney disease, confirmed that a miR-155-5p inhibitor decreases the JAK/STAT1 signaling pathway responsible for inflammation and oxidative stress in ApoE-/- diabetic mice by upregulating SOCS1 [71].

**Figure 4:**
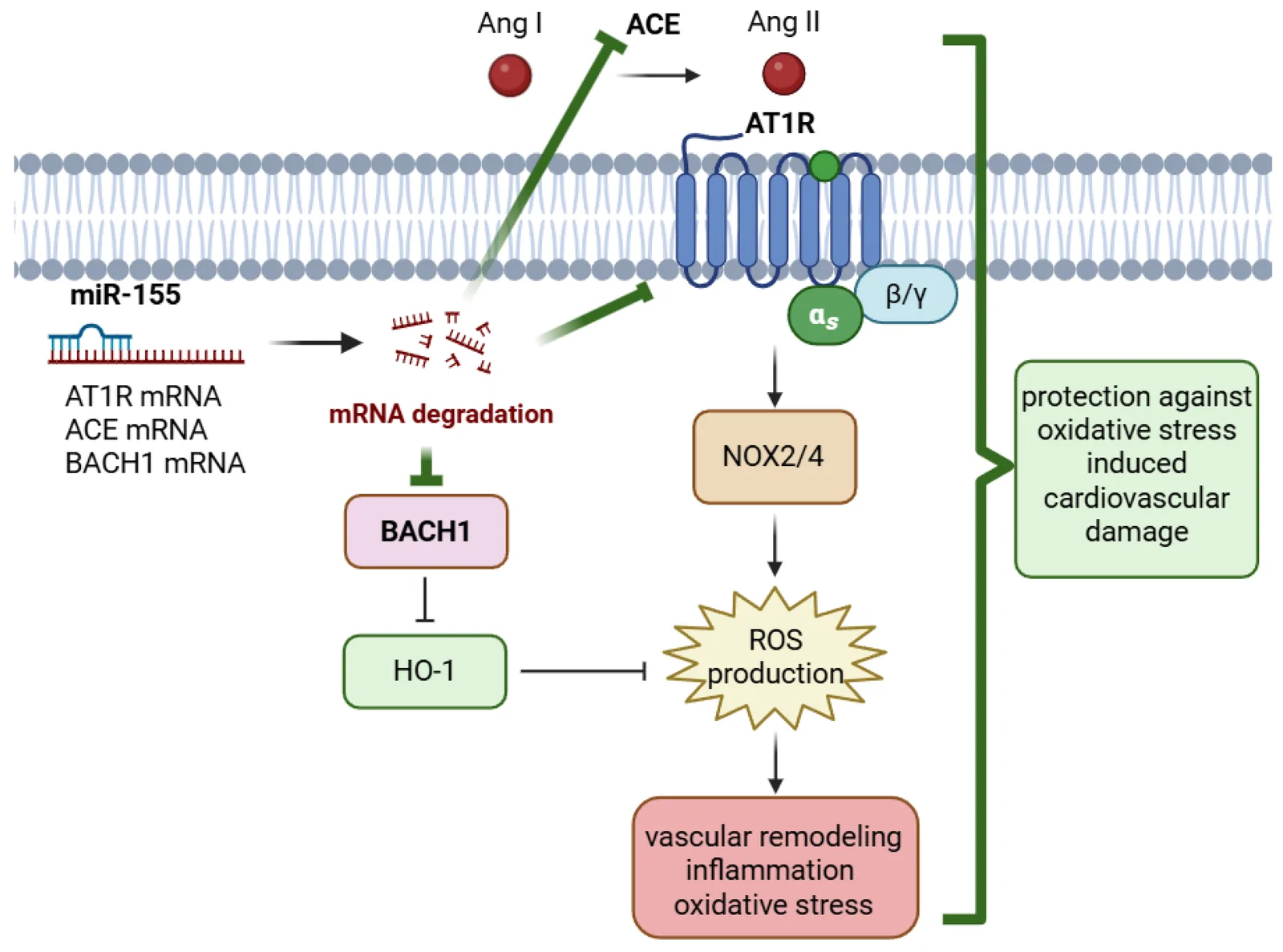
Proposed antioxidant and vasculoprotective effects of miR-155 through modulation of the renin–angiotensin system and BACH1 signaling. Increased miR-155 expression promotes the degradation of AT1R, ACE, and BACH1 mRNAs. Downregulation of AT1R attenuates Ang II-mediated signaling, reducing NOX2/4 activation, ROS generation, and downstream vascular inflammation, oxidative stress, and remodeling. In parallel, repression of BACH1 relieves its inhibitory effect on heme oxygenase-1 (HO-1), enhancing antioxidant defenses and further limiting ROS accumulation. Collectively, these mechanisms contribute to the protective role of miR-155 against oxidative stress-induced cardiovascular damage. Abbreviations: ACE: Angiotensin-converting enzyme; Ang II: angiotensin II; AT1R: angiotensin II receptor type 1; BACH1: BTB and CNC homology 1; HO-1: heme oxygenase-1; NOX2: NADPH oxidase 2; NOX4: NADPH oxidase 4; ROS: reactive oxygen species. Created in BioRender. Cacciapuoti, M. (2026) https://BioRender.com/2hlhmsy.

On the other hand, an opposite role of miR-155 in the RAAS axis and Ang II-induced oxidative stress and cardiovascular remodeling in hypertension was proposed (Figure 4). A robust proof based on functional studies comes from the paper by Dupont et al., who pointed out miR-155 as the most down-regulated miRNA in vascular aging in mice’ smooth muscle cells with intact mineralocorticoid receptors [32].

In mesenteric vessels of mice, a decrease in miR-155 was associated with a concomitant increase in MR mRNA with aging. Restoration of miR-155 in smooth muscle cells of aged MR-intact mice reduced Cav1.2, the pore-forming L-type Calcium Channel (LTCC), and Agtr1 mRNA and attenuated AT II-induced vasoconstriction and oxidative stress [32]. Wu et al. observed that in VSMC cells of spontaneously hypertensive rats, miR-155 mimic inhibited ACE expression and reduced ROS production along with IL-1β and TNF-α expression, thereby impairing VSMC migration and oxidative stress, which contribute to maladaptive vascular remodeling in hypertension and related organ damage. However, the same effect was not detected after exogenous Ang II administration, suggesting ACE could be a direct target of miR-155-5p [40]. Tong et al. confirmed the inhibitory role of miR-155 in VSMC migration and oxidative stress by reporting that exogenous miR-155-5p administration reduced NOX2 and NOX4 protein expression and activity in VSMCs from spontaneously hypertensive rats by knocking down BACH1 expression, a transcriptional repressor of the cytoprotective enzyme heme oxygenase-1 (HO-1) [35]. Conversely, BACH1, which is also expressed on chromosome 21, was not identified as a target of miR-155 in the process of senescence in endothelial cells derived from mouse aortas in the study of He et al. [33].

Bernard-Harrison et al. reported that in Dahl salt-sensitive mice under a high-salt diet, miR-155 serum and aortic levels were lower than in Dahl salt-sensitive mice under a low-salt diet, while AT1R levels were higher, together with p-MYPT1/MYPT-1 and proteinuria, suggesting a deleterious role of low levels of miR-155 [53]. In spontaneously hypertensive rats, miR-155 was instead elevated, while AT1R levels and proteinuria were decreased, suggesting that miR-155 might contribute to keeping AT1R levels low, thereby limiting Ang II-oxidative stress-mediated kidney damage despite hypertension [53]. However, no causal relationship can be inferred from these results. Song et al. observed that treatment with a soybean-derived antihypertensive (LSW) reduced oxidative stress and inflammation while increasing miR-155-5p compared to Ang-II- and control-treated HUVECs [50]. Moreover, in the context of myocardial injury, miR-155 was increased and played a regulatory role in oxidative stress-induced necrosis in cardiomyocyte progenitor cells by targeting receptor-interacting protein 1 (RIP1) [43]. Finally, treatment-based evidence shows that in HUVECs and PBMCs, miR-155 was up-regulated along with antioxidant genes’ expression as a result of hydroxyurea, an antioxidant agent prescribed in sickle-cell anemia [56].

MiR-155 was also evaluated in a broad number of small patient cohorts affected by cardiovascular diseases. However, the available evidence is largely associative, with studies reporting heterogeneous and sometimes opposing associations with oxidative stress. For example, miR-155 was observed to be up-regulated in placentas from pre-eclamptic women vs. controls and affects cysteine-rich protein 61 (CYR61), a significant premature angiogenesis component in gestation [72]. Another example is Friedrich’s ataxia patients, where a polymorphic miR-155 binding site in AGTR1 was associated with cardiac hypertrophy, while in CD14 + monocytes from patients affected by coronary disease, miR-155 expression was up-regulated [47]. Additionally, a decrease in miR-155 expression was detected with aging [73]. Serum miR-155 predicted the BP treatment response to MR antagonists in elderly humans [32]. Oxidative stress development is also associated with cigarette smoking. Interestingly, miR-155 levels were statistically increased right after smoking in a cohort of smokers [37]. Duisenbeck et al. reported that in diabetic patients, miR-155 levels were higher than in healthy subjects and positively correlated with HbA1c and glycemia. However, no significant difference was detected in diabetic patients with macrovascular complications. Intriguingly, the same authors proposed a predictive model for the diagnosis of diabetes in obese subjects, based on the combination of miR-210, miR-155,-5p glutathione peroxidase, lipid peroxidation, and BMI [59], suggesting a close relationship between obesity, diabetes, and oxidative stress. The relationship between miR-155-3p, glucose levels, and oxidative stress parameters was confirmed in a study conducted in metabolic syndrome patients [58]. Moawad et al. compared coronary heart disease patients with healthy subjects and showed higher levels of miR-155-5p along with higher MDA and lower SOD levels in the first group [60]. Furthermore, in a cohort of patients with Gitelman syndrome, plasma miR-155 levels were higher than in controls, proposing a role of this miRNA in their well-established protection against Ang II-induced cardiovascular remodeling [28]. Finally, given the location of the miR-155 gene on chromosome 21, multiple studies in Down syndrome (DS)—a population burdened by a higher prevalence of cardiovascular disease and higher risk for chemotherapy cardiotoxicity—have been conducted [21]. MiR-155 expression was not different in ex vivo studies conducted on heart samples from DS heart donors vs. non-DS heart donors. However, in non-DS donors, there was a positive association between BACH1 mRNA levels and miR-155 expression. This suggested that in the myocardium of euploid subjects, increasing levels of the inhibitor miR-155 would be necessary to target increasing levels of the BACH1 mRNA transcript. In contrast, the lack of a significant association between BACH1 mRNA and miR-155 levels in samples from donors with DS was proposed to be indicative of a disruption in this layer of control against oxidative stress in DS [67]. However, the lack of functional studies does not allow us to consider a causal relationship between miR-155 and oxidative stress in these studies.

Overall, evidence on the role of miR-155 in oxidative stress in cardiovascular disease is broad and heterogeneous, suggesting that miR-155 may function as a redox rheostat rather than as a purely pro- or antioxidant mediator. Based on the above-mentioned evidence, no certain conclusions can be drawn about a protective or harmful role of this miRNA in cardiovascular disease.

So far, only therapeutics based on miR-155 antagonists have been developed, mostly in cancer fields [15]. One example is cobomarsen, which was moved into phase II for T-cell leukemia/lymphoma [74]. Moreover, MRG-107 is an antagomir of miR-155 aimed at inhibiting the activity of miR-155 in immune mechanisms and inflammation in amyotrophic lateral sclerosis (ALS), with promising results in preclinical models [75]. In fibrosis research, local injection of antagomiR-155 was demonstrated to inhibit the Wnt/β catenin and AKT signaling pathways, thereby reducing fibrosis [76]. Regarding cardiovascular research, knocking out miR-155 in atherogenic apolipoprotein E knockout (ApoE KO, or ApoE-/-) mice resulted in the establishment of the first metabolically healthy obesity (MHO) mouse model with decreased aortic atherosclerosis, increased obesity, white adipose tissue hypertrophy, and non-alcoholic fatty liver disease but without insulin resistance [77]. On the other hand, promoting the expression of miR-155 was proposed as an effective strategy to regulate blood pressure in hypertensive disorders [15]. Nevertheless, no therapeutic strategy based on miR-155 has yet reached a clinical stage.

## 4. Conclusions and Future Directions

Although miR-103 and miR-155 are both involved in oxidative stress pathways, miR-155 emerges as a potential regulator of inflammatory-redox signaling, whereas miR-103 appears more closely linked to cell fate and metabolic pathways. In both cases, available evidence supports a context-dependent role that challenges a simplistic classification as pro- or antioxidant miRNAs.

Future studies should clarify whether the divergent pro- and antioxidant effects depend primarily on cell type, disease stage, inflammatory milieu, or compensatory adaptive responses. For example, most of the reported effects related to oxidative stress have been demonstrated in acute settings, and studies on long-standing mimic/inhibition of specific miRNAs are lacking. A substantial proportion of the available literature remains associative, with limited mechanistic validation through gain- and loss-of-function approaches. In addition, only a few validated targets of miR-155 and miR-103 within the context of oxidative stress were identified. The in vitro models of oxidative stress were mostly artificially induced via H_2_O_2_ exposure and therefore only partially mimic the real oxidative conditions of cells. Moreover, oxidative stress was assessed differently across the studies. Importantly, studies based on humans are the minority, are characterized by low standardization, and predominantly yield indirect evidence linking miR-155 to oxidative stress. Most human-based studies are on small cohorts of patients, and longitudinal clinical investigations are currently lacking. Finally, clinical studies investigating miR-103’s relationship with oxidative stress are not available yet. It should be noted that a limitation of this review is that the literature search was restricted to the PubMed database, which may have resulted in the omission of studies indexed exclusively in other databases.

The clinical development of anti-miR-103 and anti-miR-155 therapies has been pursued almost exclusively in non-cardiovascular conditions. However, to the best of our knowledge, no therapeutic approach for cardiovascular disease has yet progressed beyond the preclinical stage. The marked heterogeneity observed across the studies described in this review suggests that the biological effects of miR-103 and miR-155 in cardiovascular disorders depend on specific experimental and pathological context. These findings raise concerns about the possibility of a systemic inhibition or overexpression of miR-103 or miR-155, which could produce different, or even opposite, biological effects depending on the target tissue, cell type, and inflammatory microenvironment. Consequently, a therapeutic strategy that is beneficial in one cardiovascular setting may suppress protective responses or exacerbate pathological mechanisms in another. Future translational studies should therefore focus on developing tissue- and cell-specific delivery systems while carefully evaluating off-target effects, pharmacokinetics, dosing regimens, immunogenicity, and long-term safety. Such knowledge will not only improve our understanding of the pathophysiological role of miR-103 and miR-155 in cardiovascular disease but may also pave the way for the development of novel miRNA-based therapeutic strategies targeting oxidative stress and cardiovascular remodeling.

## Data Availability

No new data were created or analyzed in this study. Data sharing is not applicable to this article.

## References

[1] Pizzino G., Irrera N., Cucinotta M., Pallio G., Mannino F., Arcoraci V., Squadrito F., Altavilla D., Bitto A. (2017). Oxidative Stress: Harms and Benefits for Human Health. Oxid. Med. Cell. Longev..

[2] Ravarotto V., Bertoldi G., Innico G., Gobbi L., Calò L.A. (2021). The Pivotal Role of Oxidative Stress in the Pathophysiology of Cardiovascular-Renal Remodeling in Kidney Disease. Antioxidants.

[3] Basso A., Baldini P., Bertoldi G., Driussi G., Caputo I., Bettin E., Cacciapuoti M., Calò L.A. (2024). Oxidative stress reduction by icodextrin-based glucose-free solutions in peritoneal dialysis: Support for new promising approaches. Artif. Organs.

[4] Basso A., Cacciapuoti M., Stefanelli L.F., Nalesso F., Calò L.A. (2024). Glucose-Free Solutions Mediated Inhibition of Oxidative Stress and Oxidative Stress-Related Damages in Peritoneal Dialysis: A Promising Solution. Life.

[5] Sugamura K., Keaney J.F. (2011). Reactive oxygen species in cardiovascular disease. Free. Radic. Biol. Med..

[6] Pietrangelo D., Lopa C., Litterio M., Cotugno M., Rubattu S., Lombardi A. (2025). Metabolic Disturbances Involved in Cardiovascular Diseases: The Role of Mitochondrial Dysfunction, Altered Bioenergetics and Oxidative Stress. Int. J. Mol. Sci..

[7] Sgarabotto L., Ravarotto V., Stefanelli L.F., Cacciapuoti M., Davis P.A., Nalesso F., Calò L.A. (2023). Oxidants and Cardiorenal Vascular Remodeling—Insights from Rare Genetic Tubulopathies: Bartter’s and Gitelman’s Syndromes. Antioxidants.

[8] Lee Y., Im E. (2021). Regulation of miRNAs by Natural Antioxidants in Cardiovascular Diseases: Focus on SIRT1 and eNOS. Antioxidants.

[9] Abolhasani S., Ahmadi Y., Fattahi D., Rostami Y., Chollou K.M. (2025). microRNA-Mediated Regulation of Oxidative Stress in Cardiovascular Diseases. J. Clin. Lab. Anal..

[10] Ma J., Chen X. (2022). Advances in pathogenesis and treatment of essential hypertension. Front. Cardiovasc. Med..

[11] An Y., Xu B.-T., Wan S.-R., Ma X.-M., Long Y., Xu Y., Jiang Z.-Z. (2023). The role of oxidative stress in diabetes mellitus-induced vascular endothelial dysfunction. Cardiovasc. Diabetol..

[12] Yun H.R., Singh M.K., Han S., Ranbhise J.S., Ha J., Kim S.S., Kang I. (2025). Integrating Senescence and Oxidative Stress in Cardiac Disease. Int. J. Mol. Sci..

[13] Ali F., Shen A., Islam W., Saleem M.Z., Muthu R., Xie Q., Wu M., Cheng Y., Chu J., Lin W. (2022). Role of MicroRNAs and their corresponding ACE2/Apelin signaling pathways in hypertension. Microb. Pathog..

[14] Peters L.J.F., Floege J., Biessen E.A.L., Jankowski J., van der Vorst E.P.C. (2020). MicroRNAs in Chronic Kidney Disease: Four Candidates for Clinical Application. Int. J. Mol. Sci..

[15] Zhao X., Wang P. (2025). Interpretation of the molecular mechanism and therapeutic potential of microRNA-155 in cardiovascular and cerebrovascular diseases (Review). Int. J. Mol. Med..

[16] Bátkai S., Thum T. (2011). MicroRNAs in Hypertension: Mechanisms and Therapeutic Targets. Curr. Hypertens. Rep..

[17] Logan S.M., Gupta A., Wang A., Levy R.J., Storey K.B. (2021). Isoflurane and low-level carbon monoxide exposures increase expression of pro-survival miRNA in neonatal mouse heart. Cell Stress Chaperon..

[18] Qi H., Ren J., E M., Zhang Q., Cao Y., Ba L., Song C., Shi P., Fu B., Sun H. (2019). MiR-103 inhibiting cardiac hypertrophy through inactivation of myocardial cell autophagy via targeting TRPV3 channel in rat hearts. J. Cell. Mol. Med..

[19] Lu Q., Ma Z., Ding Y., Bedarida T., Chen L., Xie Z., Song P., Zou M.-H. (2019). Circulating miR-103a-3p contributes to angiotensin II-induced renal inflammation and fibrosis via a SNRK/NF-κB/p65 regulatory axis. Nat. Commun..

[20] Xu M.-C., Gao X.-F., Ruan C., Ge Z.-R., Lu J.-D., Zhang J.-J., Zhang Y., Wang L., Shi H.-M. (2015). miR-103 Regulates Oxidative Stress by Targeting the BCL2/Adenovirus E1B 19 kDa Interacting Protein 3 in HUVECs. Oxidative Med. Cell. Longev..

[21] Papadopoulos K.I., Papadopoulou A., Aw T.C. (2023). Beauty and the beast: Host microRNA-155 versus SARS-CoV-2. Hum. Cell.

[22] Nemecz M., Alexandru N., Tanko G., Georgescu A. (2016). Role of MicroRNA in Endothelial Dysfunction and Hypertension. Curr. Hypertens. Rep..

[23] Wang X., Xiong X., Jiang W., Xu S., Li J. (2025). Orientin Alleviates Oxidative Stress And Apoptosis In Diabetic Cardiomyopathy Via The Lncrna H19/Mir-103-3p/ALDH2/PI3K/AKT Axis. Arq. Bras. Cardiol..

[24] Zhang X., Yuan S., Liu J., Tang Y., Wang Y., Zhan J., Fan J., Nie X., Zhao Y., Wen Z. (2022). Overexpression of cytosolic long noncoding RNA cytb protects against pressure-overload-induced heart failure via sponging microRNA-103-3p. Mol. Ther. Nucleic Acids.

[25] Wang Y., Song X., Li Z., Liu N., Yan Y., Li T., Sun W., Guan Y., Li M., Yang Y. (2020). MicroRNA-103 Protects Coronary Artery Endothelial Cells against H_2_O_2_-Induced Oxidative Stress via BNIP3-Mediated End-Stage Autophagy and Antipyroptosis Pathways. Oxidative Med. Cell. Longev..

[26] Wang J.-X., Zhang X.-J., Li Q., Wang K., Wang Y., Jiao J.-Q., Feng C., Teng S., Zhou L.-Y., Gong Y. (2015). MicroRNA-103/107 Regulate Programmed Necrosis and Myocardial Ischemia/Reperfusion Injury Through Targeting FADD. Circ. Res..

[27] Ellis K.L., Cameron V.A., Troughton R.W., Frampton C.M., Ellmers L.J., Richards A.M. (2013). Circulating microRNAs as Candidate Markers to Distinguish Heart Failure in Breathless Patients. Eur. J. Heart Fail..

[28] Cacciapuoti M., Caputo I., Driussi G., Davis P.A., Nalesso F., Calò L.A. (2026). Higher expression of miR-155 and miR-103 in patients with Gitelman’s syndrome: Potential implications for Angiotensin II-induced cardiovascular remodeling in hypertension. Kidney Blood Press. Res..

[29] Zhang M., Tang Y., Tang E., Lu W. (2020). MicroRNA-103 represses hepatic de novo lipogenesis and alleviates NAFLD via targeting FASN and SCD1. Biochem. Biophys. Res. Commun..

[30] Huang Y. (2017). Preclinical and Clinical Advances of GalNAc-Decorated Nucleic Acid Therapeutics. Mol. Ther. Nucleic Acids.

[31] Ge X., Meng Q., Liu J., Wang E., Liu X., Shi S., Gong X., Liu Z., Han W., Zhou X. (2026). Cardiac extracellular vesicles aggravate cardiomyocyte ferroptosis in myocardial ischemia-reperfusion injury via miR-155-5p-Nfe2l2 signaling. Biochim. Biophys. Acta (BBA)-Gen. Subj..

[32] DuPont J.J., McCurley A., Davel A.P., McCarthy J., Bender S.B., Hong K., Yang Y., Yoo J.-K., Aronovitz M., Baur W.E. (2016). Vascular mineralocorticoid receptor regulates microRNA-155 to promote vasoconstriction and rising blood pressure with aging. J. Clin. Investig..

[33] He J., Zhang B., Zhang H., Tu Q., Chen X., Qiu Y., Liu Z., Xia W., Wu X., Tao J. (2025). M1 Macrophage is a Novel Potential Trigger for Endothelial Senescence: Role of Exosomal miR-155 Targeting SOCS1 Signal. Hum. Mutat..

[34] Zhang Y., Hei F., Xiao Y., Liu Y., Han J., Hu D., Wang H. (2024). Acidic fibroblast growth factor inhibits reactive oxygen species-induced epithelial–mesenchymal transdifferentiation in vascular endothelial cells via the miR-155-5p/SIRT1/Nrf2/HO-1 pathway to promote wound healing in diabetic mice. Burn. Trauma.

[35] Tong Y., Zhou M.-H., Li S.-P., Zhao H.-M., Zhang Y.-R., Chen D., Wu Y.-X., Pang Q.-F. (2023). MiR-155-5p Attenuates Vascular Smooth Muscle Cell Oxidative Stress and Migration via Inhibiting BACH1 Expression. Biomedicines.

[36] Zhao M., Lei J., Deng F., Zhao C., Xu T., Ji B., Fu M., Wang X., Sun M., Zhang M. (2024). Gestational Hypoxia Impaired Endothelial Nitric Oxide Synthesis Via miR-155-5p/NADPH Oxidase/Reactive Oxygen Species Axis in Male Offspring Vessels. J. Am. Heart Assoc..

[37] Frati G., Forte M., di Nonno F., Bordin A., Chimenti I., Picchio V., Cavarretta E., Stanzione R., Bianchi F., Carnevale R. (2020). Inhibition of miR-155 Attenuates Detrimental Vascular Effects of Tobacco Cigarette Smoking. J. Am. Heart Assoc..

[38] He X., Wang Z., Wei L., Cheng X., Chen L., Gao F., Jiang H. (2020). Indoxyl sulfate promotes osteogenic differentiation of vascular smooth muscle cells by miR-155-5p-dependent downregulation of matrix Gla protein via ROS/NF-κB signaling. Exp. Cell Res..

[39] Wang X., Han W., Zhang Y., Zong Y., Tan N., Zhang Y., Li L., Liu C., Liu L. (2022). Soluble Epoxide Hydrolase Inhibitor t-AUCB Ameliorates Vascular Endothelial Dysfunction by Influencing the NF-κB/miR-155-5p/eNOS/NO/IκB Cycle in Hypertensive Rats. Antioxidants.

[40] Wu N., Ye C., Zheng F., Wan G.-W., Wu L.-L., Chen Q., Li Y.-H., Kang Y.-M., Zhu G.-Q. (2020). MiR155-5p Inhibits Cell Migration and Oxidative Stress in Vascular Smooth Muscle Cells of Spontaneously Hypertensive Rats. Antioxidants.

[41] Jia C., Chen H., Wei M., Chen X., Zhang Y., Cao L., Yuan P., Wang F., Yang G., Ma J. (2017). Gold nanoparticle-based miR155 antagonist macrophage delivery restores the cardiac function in ovariectomized diabetic mouse model. Int. J. Nanomed..

[42] Liu Y., Pan Q., Zhao Y., He C., Bi K., Chen Y., Zhao B., Chen Y., Ma X. (2015). MicroRNA-155 Regulates ROS Production, NO Generation, Apoptosis and Multiple Functions of Human Brain Microvessel Endothelial Cells Under Physiological and Pathological Conditions. J. Cell. Biochem..

[43] Liu J., van Mil A., Vrijsen K., Zhao J., Gao L., Metz C.H.G., Goumans M.-J., Doevendans P.A., Sluijter J.P.G. (2010). MicroRNA-155 prevents necrotic cell death in human cardiomyocyte progenitor cells via targeting RIP1. J. Cell. Mol. Med..

[44] Wang F., Zhang J., Niu G., Weng J., Zhang Q., Xie M., Li C., Sun K. (2022). Apigenin inhibits isoproterenol-induced myocardial fibrosis and Smad pathway in mice by regulating oxidative stress and miR-122-5p/155-5p expressions. Drug Dev. Res..

[45] Sun H.-J., Zhao M.-X., Liu T.-Y., Ren X.-S., Chen Q., Li Y.-H., Kang Y.-M., Zhu G.-Q. (2016). Salusin-β induces foam cell formation and monocyte adhesion in human vascular smooth muscle cells via miR155/NOX2/NFκB pathway. Sci. Rep..

[46] Yang Z., Zheng B., Zhang Y., He M., Zhang X.-H., Ma D., Zhang R.-N., Wu X.-L., Wen J.-K. (2015). miR-155-dependent regulation of mammalian sterile 20-like kinase 2 (MST2) coordinates inflammation, oxidative stress and proliferation in vascular smooth muscle cells. Biochim. Biophys. Acta (BBA)-Mol. Basis Dis..

[47] Tian F.-J., An L.-N., Wang G.-K., Zhu J.-Q., Li Q., Zhang Y.-Y., Zeng A., Zou J., Zhu R.-F., Han X.-S. (2014). Elevated microRNA-155 promotes foam cell formation by targeting HBP1 in atherogenesis. Cardiovasc. Res..

[48] Kim J., Lee K.-S., Kim J.-H., Lee D.-K., Park M., Choi S., Park W., Kim S., Choi Y.K., Hwang J.Y. (2017). Aspirin prevents TNF-α-induced endothelial cell dysfunction by regulating the NF-κB-dependent miR-155/eNOS pathway: Role of a miR-155/eNOS axis in preeclampsia. Free. Radic. Biol. Med..

[49] Kim T.-H., Kim J.-Y., Bae J., Kim Y.-M., Won M.-H., Ha K.-S., Kwon Y.-G., Kim Y.-M. (2021). Korean Red ginseng prevents endothelial senescence by downregulating the HO-1/NF-κB/miRNA-155-5p/eNOS pathway. J. Ginseng Res..

[50] Song T., Lv M., Zhou M., Huang M., Zheng L., Zhao M. (2021). Soybean-Derived Antihypertensive Peptide LSW (Leu–Ser–Trp) Antagonizes the Damage of Angiotensin II to Vascular Endothelial Cells through the Trans-vesicular Pathway. J. Agric. Food Chem..

[51] Xiong M., Jia C., Cui J., Wang P., Du X., Yang Q., Zhu Y., Wang W., Zhang T., Chen Y. (2015). Shexiang Tongxin dropping pill attenuates atherosclerotic lesions in ApoE deficient mouse model. J. Ethnopharmacol..

[52] Cai R., Hao Y., Liu Y.-Y., Huang L., Yao Y., Zhou M.-S. (2020). Tumor Necrosis Factor Alpha Deficiency Improves Endothelial Function and Cardiovascular Injury in Deoxycorticosterone Acetate/Salt-Hypertensive Mice. BioMed Res. Int..

[53] Harrison-Bernard L.M., Raij L., Tian R.X., Jaimes E.A. (2024). Genetically conditioned interaction among microRNA-155, alpha-klotho, and intra-renal RAS in male rats: Link to CKD progression. Physiol. Rep..

[54] Costantino S., Paneni F., Lüscher T.F., Cosentino F. (2015). MicroRNA profiling unveils hyperglycaemic memory in the diabetic heart. Eur. Heart J..

[55] Muñoz-Pacheco P., Ortega-Hernández A., Miana M., Cachofeiro V., Fernández-Cruz A., Gómez-Garre D. (2012). Ezetimibe inhibits PMA-induced monocyte/macrophage differentiation by altering microRNA expression: A novel anti-atherosclerotic mechanism. Pharmacol. Res..

[56] Santana S.S., Pitanga T.N., de Santana J.M., Zanette D.L., Vieira J.d.J., Yahouédéhou S.C.M.A., Adanho C.S.A., Viana S.d.M., Luz N.F., Borges V.M. (2020). Hydroxyurea Scavenges Free Radicals and Induces the Expression of Antioxidant Genes in Human Cell Cultures Treated with Hemin. Front. Immunol..

[57] Nguyen D.D.N., Zain S.M., Kamarulzaman M.H., Low T.Y., Chilian W.M., Pan Y., Ting K.N., Hamid A., Kadir A.A., Pung Y.-F. (2021). Intracellular and exosomal microRNAome profiling of human vascular smooth muscle cells during replicative senescence. Am. J. Physiol. Circ. Physiol..

[58] Khedr N.F., Zahran E.S., Ebeid A.M., Melek S.T., Werida R.H. (2024). Effect of green coffee on miR-133a, miR-155 and inflammatory biomarkers in obese individuals. Diabetol. Metab. Syndr..

[59] Duisenbek A., Pérez M.D.A., Pérez M., Benitez J.M.A., Pérez V.R.P., Ortega J.G., Ussipbek B., Yessenbekova A., López-Armas G.C., Ablaikhanova N. (2024). Unveiling the Predictive Model for Macrovascular Complications in Type 2 Diabetes Mellitus: microRNAs Expression, Lipid Profile, and Oxidative Stress Markers. Int. J. Mol. Sci..

[60] Moawad A.M., Awady S., Ali A.A.E.R., Abdelgwad M., Belal S., Taha S.H.N., Mohamed M.I., Hassan F.M. (2024). Phthalate Exposure and Coronary Heart Disease: Possible Implications of Oxidative Stress and Altered miRNA Expression. Chem. Res. Toxicol..

[61] Saghati A.A., Sharifi Z., Hatamikhah M., Salimi M., Talkhabi M. (2024). Unraveling the relevance of SARS-CoV-2 infection and ferroptosis within the heart of COVID-19 patients. Heliyon.

[62] Kelly M., Bagnall R.D., Peverill R.E., Donelan L., Corben L., Delatycki M.B., Semsarian C. (2011). A polymorphic miR-155 binding site in AGTR1 is associated with cardiac hypertrophy in Friedreich ataxia. J. Mol. Cell. Cardiol..

[63] Kim S.-M., Lim S.-M., Yoo J.-A., Woo M.-J., Cho K.-H. (2015). Consumption of high-dose vitamin C (1250 mg per day) enhances functional and structural properties of serum lipoprotein to improve anti-oxidant, anti-atherosclerotic, and anti-aging effects via regulation of anti-inflammatory microRNA. Food Funct..

[64] Chen H., Gao M.Y.L., Zhang L., He F.L., Shi Y.K., Pan X.H., Wang H. (2019). MicroRNA-155 affects oxidative damage through regulating autophagy in endothelial cells. Oncol. Lett..

[65] Liu M., Saredy J., Zhang R., Shao Y., Sun Y., Yang W.Y., Wang J., Liu L., Drummer C., Johnson C. (2020). Approaching Inflammation Paradoxes—Proinflammatory Cytokine Blockages Induce Inflammatory Regulators. Front. Immunol..

[66] Constantin A., Comarița I.K., Alexandru N., Filippi A., Bojin F., Gherghiceanu M., Vîlcu A., Nemecz M., Niculescu L.S., Păunescu V. (2022). Stem cell-derived extracellular vesicles reduce the expression of molecules involved in cardiac hypertrophy—In a model of human-induced pluripotent stem cell-derived cardiomyocytes. Front. Pharmacol..

[67] Hefti E., Quiñones-Lombraña A., Redzematovic A., Hui J., Blanco J.G. (2014). Analysis of mtDNA, *miR-155* and *BACH1* expression in hearts from donors with and without Down syndrome. Mitochondrial DNA Part A.

[68] Jia C., Xiong M., Wang P., Cui J., Du X., Yang Q., Wang W., Chen Y., Zhang T. (2014). Notoginsenoside R1 Attenuates Atherosclerotic Lesions in ApoE Deficient Mouse Model. PLoS ONE.

[69] Liu D., Han L., Wu X., Yang X., Zhang Q., Jiang F. (2014). Genome-wide microRNA changes in human intracranial aneurysms. BMC Neurol..

[70] Witvrouwen I., Mannaerts D., Ratajczak J., Boeren E., Faes E., Van Craenenbroeck A.H., Jacquemyn Y., Van Craenenbroeck E.M. (2021). MicroRNAs targeting VEGF are related to vascular dysfunction in preeclampsia. Biosci. Rep..

[71] Prieto I., Kavanagh M., Jimenez-Castilla L., Pardines M., Lazaro I., del Real I.H., Flores-Muñoz M., Egido J., Lopez-Franco O., Gomez-Guerrero C. (2023). A mutual regulatory loop between miR-155 and SOCS1 influences renal inflammation and diabetic kidney disease. Mol. Ther. Nucleic Acids.

[72] Zhang Y., Diao Z., Su L., Sun H., Li R., Cui H., Hu Y. (2010). MicroRNA-155 contributes to preeclampsia by down-regulating CYR61. Am. J. Obstet. Gynecol..

[73] Hooten N.N., Abdelmohsen K., Gorospe M., Ejiogu N., Zonderman A.B., Evans M.K. (2010). microRNA Expression Patterns Reveal Differential Expression of Target Genes with Age. PLoS ONE.

[74] Anastasiadou E., Seto A.G., Beatty X., Hermreck M., Gilles M.-E., Stroopinsky D., Pinter-Brown L.C., Pestano L., Marchese C., Avigan D. (2021). Cobomarsen, an Oligonucleotide Inhibitor of miR-155, Slows DLBCL Tumor Cell Growth In Vitro and In Vivo. Clin. Cancer Res..

[75] Seyhan A.A. (2024). Trials and Tribulations of MicroRNA Therapeutics. Int. J. Mol. Sci..

[76] Yan Q., Chen J., Li W., Bao C., Fu Q. (2016). Targeting miR-155 to Treat Experimental Scleroderma. Sci. Rep..

[77] Virtue A., Johnson C., Lopez-Pastraña J., Shao Y., Fu H., Li X., Li Y.-F., Yin Y., Mai J., Rizzo V. (2017). MicroRNA-155 Deficiency Leads to Decreased Atherosclerosis, Increased White Adipose Tissue Obesity, and Non-alcoholic Fatty Liver Disease. J. Biol. Chem..

